# Sensitivity and specificity of current diagnostic guidelines in children with macrophage activation syndrome complicating systemic juvenile idiopathic arthritis

**DOI:** 10.1186/1546-0096-9-S1-P101

**Published:** 2011-09-14

**Authors:** Sergio Davì, Bianca Lattanzi, Silvia Rosina, Erkan Demirkaya, Nicolino Ruperto, Alberto Martini, Randy Q Cron, Angelo Ravelli

**Affiliations:** 1 (For the) Investigator Consortium for MAS Diagnostic Guidelines

## Background

Early diagnosis of macrophage activations syndrome (MAS) in systemic juvenile idiopathic arthritis (sJIA) may be challenging because it may mimic the clinical features of the underlying disease or be confused with an infectious complication. However, the diagnostic value of the guidelines for hemophagocytic lymphohistiocytosis (HLH) (1) or sJIA-associated MAS (2) has seldom been examined.

## Objective

To investigate the sensitivity and specificity of diagnostic guidelines for HLH and sJIA-associated MAS in patients with sJIA who developed MAS.

## Methods

The study sample included 155 children with sJIA who had MAS (diagnosed and treated as such by the attending physician) and 2 control groups with potentially “confusable” conditions, including active sJIA without MAS (n=303) and a systemic febrile infection requiring hospitalization (n=191). Diagnostic guidelines for HLH and sJIA-associated MAS were applied to all MAS and control patients. Because no patient had NK-cell activity and soluble CD25 determination available and bone marrow aspirate was performed in only a few patients, these 3 criteria were excluded from HLH guidelines. HLH criteria were, therefore, met when at least 4 of the 5 remaining variables were present. sJIA-associated MAS criteria were met when at least 2 laboratory criteria or at least 1 laboratory criterion and 1 clinical criterion were present. Sensitivity and specificity of guidelines in discriminating patients with MAS from control patients were assessed.

## Results

The table shows the comparison of sensitivity and specificity of diagnostic guidelines.

**Table:** 

	MAS vs. active sJIA		MAS vs. systemic infection	
	
Diagnostic guidelines	Sensitivity	Specificity	Sensitivity	Specificity
HLH	0.26	1	0.26	0.98
sJIA-associated MAS	0.87	0.91	0.87	0.85

## Conclusions

The diagnostic guidelines for sJIA-associated MAS revealed strong sensitivity and specificity, whereas HLH guidelines were highly specific, but lacked sensitivity. Sensitivity of HLH was mostly hampered by the excessive stringent threshold for cytopenia and hypofibrinogenemia, and the infrequent occurrence of splenomegaly in patients with MAS.
